# A Critical Review of the Bacterial Baptism Hypothesis and the Impact of Cesarean Delivery on the Infant Microbiome

**DOI:** 10.3389/fmed.2018.00135

**Published:** 2018-05-04

**Authors:** Lisa F. Stinson, Matthew S. Payne, Jeffrey A. Keelan

**Affiliations:** Division of Obstetrics and Gynaecology, Faculty of Health and Medical Sciences, The University of Western Australia, Perth, WA, Australia

**Keywords:** cesarean delivery, vaginal delivery, delivery mode, neonatal microbiome, infant microbiome, developmental origins, vaginal seeding

## Abstract

Numerous studies suggest that infants delivered by cesarean section are at a greater risk of non-communicable diseases than their vaginal counterparts. In particular, epidemiological studies have linked Cesarean delivery with increased rates of asthma, allergies, autoimmune disorders, and obesity. Mode of delivery has also been associated with differences in the infant microbiome. It has been suggested that these differences are attributable to the “bacterial baptism” of vaginal birth, which is bypassed in cesarean deliveries, and that the abnormal establishment of the early-life microbiome is the mediator of later-life adverse outcomes observed in cesarean delivered infants. This has led to the increasingly popular practice of “vaginal seeding”: the iatrogenic transfer of vaginal microbiota to the neonate to promote establishment of a “normal” infant microbiome. In this review, we summarize and critically appraise the current evidence for a causal association between Cesarean delivery and neonatal dysbiosis. We suggest that, while Cesarean delivery is certainly associated with alterations in the infant microbiome, the lack of exposure to vaginal microbiota is unlikely to be a major contributing factor. Instead, it is likely that indication for Cesarean delivery, intrapartum antibiotic administration, absence of labor, differences in breastfeeding behaviors, maternal obesity, and gestational age are major drivers of the Cesarean delivery microbial phenotype. We, therefore, call into question the rationale for “vaginal seeding” and support calls for the halting of this practice until robust evidence of need, efficacy, and safety is available.

## Background

Cesarean section (CS) delivery can be a life-saving procedure. However, globally, rates of elective CS delivery are increasing. In Organization for Economic Co-operation and Development countries, more than a quarter of infants are now born by CS (1). The increasing popularity of elective CS delivery has coincided with a rising prevalence in non-communicable diseases, prompting investigations into a possible causal link between the two.

Epidemiological studies have identified CS delivery as a risk factor for neonatal infections. In the US, 64–82% of reported cases of neonatal methicillin-resistant *Staphylococcus aureus* skin infections occur in Cesarean section delivered (CSD) infants (2). CS delivery has also been recognized as a risk factor for necrotizing enterocolitis in preterm infants (3). Epidemiological studies also suggest that CSD infants experience an increased risk of the onset of several chronic immune diseases later in life: a large body of research has linked CS delivery with childhood asthma (4–6), atopic disease (7–9), allergies (9, 10), obesity (11), type 1 diabetes mellitus (12), and inflammatory bowel disease (5, 13). More recently, CS delivery has been associated with impaired cognitive abilities in school aged children (14). It has been widely promulgated that such associations are caused by perturbations in the early-life microbiome as a consequence of lack of exposure to vaginal microbiota at delivery. However, such studies are purely associative and are unable to determine causation or mechanism. There is, therefore, an urgent need to assess whether these associations are causal or reflective of confounding factors, and to determine the extent to which the establishment of the early-life microbiome is involved. In particular, it is necessary to assess whether such studies support the rationale for “vaginal seeding” (the transfer of vaginal fluid, and the microbes contained therein, to the neonate).

The infant microbiota is foundational for later-life health (15). The early-life gut microbiota interact—primarily *via* the production and release of microbial metabolites and degradation products—with the developing immune system and contribute to immune programming and sensitization (16, 17). Aberrations in the establishment of this ecosystem in infants and children underpin the risk for immune-mediated diseases, such as asthma (18–21), allergies (22–25), and inflammatory bowel disease (26, 27), as well as metabolic conditions, such as obesity (28) and a variety of behavioral, cognitive, and mental health conditions (29). It has been suggested that a decline in exposure to microbes during prenatal and early postnatal life has contributed to the increase in the prevalence of non-communicable diseases in developed nations—the “hygiene hypothesis” (30). Thus, any effect of CS delivery on the developing infant microbiome might confer risks for later-life morbidity.

In this review, we present a critical evaluation of the current evidence for a causal link between CS delivery and neonatal dysbiosis. For the purposes of this review, we use the term “neonate” to refer to infants aged up to 3 days, and the term “infant” to refer to infants aged 4 days to 1 year.

## CS Delivery: Effects on the Neonatal Microbiome

It is commonly assumed that passage through the birth canal seeds the neonatal microbiome with vaginal bacteria. If this is so, the effect of CS delivery would be expected to be most apparent in the first days following birth, with VD neonates exhibiting higher rates of colonization with predominant vaginal bacteria, particularly specific *Lactobacillus* spp., such as *L. crispatus, L. gasseri, L. jensenii*, and *L. iners*. Such differences should be most obvious in the neonatal skin and oral microbiomes, though some effect on the gut microbiome may also be apparent. As time progresses, the effect of delivery mode would be expected to become less obvious, as other microbiome-driving factors, such as breastfeeding and environmental exposures come in to play and each microbial niche acquires its own unique microbiota.

Previous studies have reported that differences in the gut microbiome between CSD and VD neonates do not develop until several days after birth (31). Wampach et al. tracked the colonization and succession of bacteria, archaea, and microeukaryotes in the feces of infants born from vaginal (*n* = 8) or CS delivery (*n* = 7) (31). They found that differences in the gut microbiomes of CSD and VD neonates were not apparent until day 5 of life. After this time point, they reported depletion of sequences affiliated with the phylum Bacteroidetes in CSD infants, resulting in a significant increase in the Firmicutes/Bacteroidetes ratio between days 5 and 150 compared to VD infants. Considering the large number of genera and species contained within these two phyla and associated differences in 16S rRNA gene copy number, however, the biological relevance of this finding is difficult to state.

Numerous studies describing the bacterial microbiota of first pass meconium (the first fecal material, passed shortly after birth) support the notion that CSD and VD neonates do not differ in their bacterial microbiomes in the first few days following birth. Hu et al.’s study of 23 neonates (10 of which were delivered by CS), reported no significant differences in the meconium microbiota of full-term VD and CSD neonates (32). Similarly, Mshvildadze et al. reported no significant differences in the meconium microbiota of pre-term (23–32 weeks of gestational age) VD and CSD neonates (CSD *n* = 13, VD *n* = 10) (33). Dong et al. compared the vaginal and placental microbiomes of mothers, and meconium microbiomes of neonates from one Cesarean delivery and one vaginal delivery (34). There were no significant differences in bacterial communities in the meconium of the two neonates, which were similar to the mother’s placenta regardless of the method of delivery. However, given that both meconium and the placenta contain a very low biomass microbiome, this similarity may in fact be a reflection of the “kitome” [reagent-derived contaminating bacterial deoxyribonucleic acid (DNA)] (35, 36). In a more recent and much larger study, Chu et al. compared the microbial composition of 102 VD and 52 CSD neonates at birth across multiple body sites (37). Although some minor differences in the oral, nares, and skin microbial communities were observed, no differences were detected in the community structure or function of the meconium microbiome.

On the other hand, there are studies that report data to the contrary. In their investigation of the effect of gestational age on the neonatal microbiome, Ardissone et al. found that delivery mode had a strong effect on meconium microbiome structure (38). In particular, they identified four genera that were associated with mode of delivery: *Leuconostoc* spp., *Negavicoccus* spp., *Vagococcus* spp., and *Butyrivibrio* spp. Each of these genera were found to have a significantly greater relative abundance in CSD neonates (*p* < 0.05). Neonates are susceptible to *Leuconostoc* bacteremia (39). Of the other three genera noted, *Vagococcus* have been identified in wounds and infected oral habitats, but none are biologically relevant in either the neonatal or the adult human gut (40, 41). Thus their recovery might reflect contamination or incorrect OTU assignment. Importantly, no differences in relative abundances of vaginal bacteria were reported between groups. In their investigation into the meconium microbiome of Chinese neonates, Shi et al. found that delivery mode was the most significant contributing factor to the composition of the neonatal microbiome (42). They found that 16S rRNA gene amplicons generated from meconium DNA of VD infants (*n* = 8) harbored more sequences associated with Actinobacteria, Gammaproteobacteria, and Betaproteobacteria, while the meconium of CSD infants (*n* = 10) contained more sequences associated with Deinococcus, Alphaproteobacteria, and Bacilli. The authors also went on to demonstrate differences in predicted metabolic pathways, although given that their study could only detect bacterial DNA, not living bacteria *per se*, such metabolomic differences are merely speculative.

An older study by Biasucci et al. used targeted polymerase chain reaction (PCR) coupled with gradient gel electrophoresis to identify differences in colonization of selected *Bifidobacterium* spp., *Ruminococcus* spp., and *Bacteroides* spp. in VD (*n* = 23) and CSD (*n* = 23) neonates at day 3 of life (43). They found that *Bifidobacterium* spp. and *Bacteroides* spp. were detected in 56.5%, and 8.7% of VD infants, respectively, but were absent in CSD infants.

Dominguez-Bello et al. compared several body site microbiomes from VD (*n* = 4) and CSD (*n* = 6) neonates within an hour of delivery (44). The very small sample size and considerable variability in maternal vaginal microbiome are a significant limitation in this study. Nevertheless, they found that the microbiome of these neonates was homogenous across body sites, regardless of delivery mode. Given the likely importance of amniotic fluid in pre-natal microbiome seeding (45), this finding is not surprising. Unfortunately, the authors did not investigate placental or amniotic fluid microbiomes from these mothers, as these may have been the true source of the neonatal microbiome. The authors further reported that VD neonates harbored microbiomes most similar in composition to their mother’s vaginal microbiome, while CSD neonates harbored microbiomes most similar in composition to their mother’s skin. Given that neonates were swabbed within seconds of delivery, and thus it would be coated with vaginal fluids, this result is hardly surprising. This does not necessarily demonstrate colonization, however. It would have been far more informative if these authors sampled at a later time point or followed the colonization patterns of these neonates in the first few days following delivery to confirm true colonization.

Given recent evidence that microbiome colonization begins *in utero* (45, 46), the “bacterial baptism” of vaginal birth might not be as important to microbiome establishment as previously assumed. In fact, Martin et al. in a small study of five CSD and five VD neonates, demonstrated that the majority of VD infants do not acquire their mother’s vaginal lactobacilli through vertical transmission, and that *Lactobacillus* populations do not differ in the infant gut according to mode of delivery (47). Conversely, Nagpal et al. reported a lower detection rate of lactobacilli in the intestinal tracts of CSD neonates than in VD neonates using targeted real-time qualitative polymerase chain reaction (RT-qPCR) (48). Although this study also suffered from low numbers of CSD neonates (*n* = 17) compared to VD neonates (*n* = 134), its use of RNA-based methods is a strength as detection should be indicative of live bacterial cells. In a more recent study by Sakwinska et al., minimal overlap between maternal vaginal microbiota and neonatal fecal and nasal microbiota was reported at 3 days of life, regardless of delivery mode (VD *n* = 34, CSD *n* = 8) (49). The authors concluded that the transfer of maternal vaginal microbes do not play a major role in seeding neonatal microbiota.

Interestingly, very few studies have explored the relationship between the vaginal microbiome and the neonatal gut microbiome at the species- or strain-level. This is critical, as the bacterial baptism hypothesis would predict that the infant gut would be transiently colonized with the common vaginal lactobacilli (*L. crispatus, L. gasseri, L. iners*, and *L. jensenii*) and other vaginal commensals (e.g., *G. vaginalis, A. vaginae*), as opposed to other species of lactobacilli which are common in the adult gut. In particular, merely reporting on *Lactobacillus* levels fails to provide suitable resolution to assess the evidence for vertical transmission. In the aforementioned study by Nagpal et al., the absolute abundance of lactobacilli was lower in CSD vs. VD neonates; however, of the common vaginal species, only *L. gasseri* was detected in the neonates (CSD vs. VD: 6 vs. 31%), the other five species being typical gut colonizers but not found in vaginal microbiota.

While the majority of evidence suggests that delivery mode does not shape the microbiome in the first days of life, most of the studies described here involved small numbers of participants and differed substantially in the methodologies used to generate microbial profiles from samples (Table 1). Of particular importance, numerous studies did not appropriately control for contaminating reagent-derived bacterial DNA (“kitome”) that has now been shown to be ubiquitous in DNA extraction and molecular biology reagents (35). Further, appropriately controlled studies enlisting large cohorts are required to assess whether CS delivery truly disturbs the neonatal microbiome from the time of birth.

**Table 1:** Summary of the methodological approaches used by studies that have compared infant microbiota profiles between cesarean section (CS) and vaginal deliveries.

Study	*N*[cesarean section delivered (CSD)]	Bead beating step?	Gene target/s	Sequencing platform/bioinformatics pipeline	16S rRNA gene region	Primer coverage (%)^a^	Major genera with poor coverage	Negative controls used?	Conclusion
**Neonatal studies**									
Ardissone et al. (38)	52 (33)	Yes	16S rRNA	Ion Torrent PGM/Bioinformatics performed manually	V4	86.3	None	Yes^f^	Delivery mode has an effect on the gut microbiome of preterm neonates

Biasucci et al. (43)	46 (23)	Not specified	Various^c^	Targeted qualitative polymerase chain reaction (qPCR)	Various	N/A	N/A	No	Mode of delivery is associated with differences in the gut microbiota composition in neonates at day 3 of life

Chu et al. (37)	157 (52)	Yes	16S rRNA	454/QIIME v1.9.0	V5–V3	67.7	*Bifidobacterium*: 0.6% *Escherichia*/*Shigella*: 0.8% *Enterobacter*: 0%	No	Minor differences in the microbial communities of CSD neonates in some body sites, but not in meconium

Dominguez-Bello et al. (44)	10 (6)	Yes	16 S rRNA	454/Bioinformatics performed manually	V2	59.7	*Bifidobacterium*: 15% *Escherichia*/*Shigella*: 23% *Enterobacter*: 21%	No	Mode of delivery is a strong determinant of the skin, oral, and gut microbiota composition in neonates

Dong et al. (34)	2 (1)	No	16S rRNA	Genome sequencer FLX/Mothur	V3–V5	83.1	*Bifidobacterium*: 0.6%	No	Similar bacterial communities were found in the meconium of CSD and vaginally delivered (VD) neonates

Hu et al. (32)	23 (10)	No	16S rRNA	Pacbio RS/QIIME v1.5.0	V3–V4	77.7	None	No	Meconium microbiota is not affected by the mode of delivery

Mshvildadze et al. (33)	23 (13)	No	16S rRNA	454/RDP pyrosequencing pipeline	V1–V2	59.7	*Bifidobacterium*: 15% *Escherichia*/*Shigella*: 23% *Enterobacter*: 21%	No	Meconium microbiota is not affected by the mode of delivery

Sakwinska et al. (49)	42 (8)	Not specified	16S rRNA	454/QIIME v1.8.0	V4–V6	Primers not specified	Primers not specified	No	Although the nasal and gut microbiomes of CSD and VD neonates vary, this is not due to transfer of maternal vaginal microbes

Shi et al. (42)	18 (10)	No	16S rRNA	Illumina Hiseq2500/Bioinformatics performed manually	Primers not specified	N/A	N/A	Yes^f^	Mode of delivery is associated with differences in the gut microbiota composition in neonates at day 1 of life

Wampach et al. (31)	15 (7)	Yes	16S rRNA, 18S rRNA	Illumina MiSeq/LotuS	V4	86.4	None	No^e^	CSD infants experienced a delay in microbial colonization and succession in the gut, which began after day 5 of life

**Infant studies**									
Azad et al. (50)	24 (6)	Yes	16S rRNA	Illumina SI-Seq/QIIME v1.6.0	V5–V7	56.5^b^	*Bifidobacterium*: 36% *Bacteroides*: 32%	No	Mode of delivery is a strong determinant of the gut microbiota composition in infants

Bäckhed et al (51)	98 (15)	Yes	16S rRNA	Illumina Hiseq2000/Bioinformatics performed manually	Primers not specified	Primers not specified	Primers not specified	No	Mode of delivery is a strong determinant of the gut microbiota composition in infants

Chu et al. (37)	60 (22)	Yes	16S rRNA	454/QIIME v1.9.0	V5–V3	67.7	*Bifidobacterium*: 0.6% *Escherichia*/*Shigella*: 0.8% *Enterobacter*: 0%	No	By 6 weeks of age no differences between the skin, oral, nares, or gut microbiota of CSD and VD infants

Penders et al. (52)	1,032 (108)	Yes	Various^d^	Targeted qPCR	Various	N/A	N/A	No	Mode of delivery is a strong determinant of the gut microbiota composition in infants

Sakwinska et al. (49)	42 (8)	Not specified	16 S rRNA	454/QIIME v1.8.0	V4–V6	Primers not specified	Primers not specified	No	Although the nasal and gut microbiomes of CSD and VD infants vary, this is not due to transfer of maternal vaginal microbes

*^a^For domain bacteria, identified using Silva TestPrime*.

*^b^Primers contained too many ambiguities to be analyzed with Silva TestPrime. RDP probe match was used instead*.

*^c^Targeted to Bifidobacterium spp., Ruminococcus spp., and Bacteroides spp*.

*^d^Targeted to Bifidobacterium spp., E. coli, C. difficile, B. fragilis, and Lactobacillus spp*.

*^e^No extraction controls used, but low yield and negative amplicon samples were discarded*.

*^f^Negative extraction controls used for amplification, but not for sequencing*.

## CS Delivery: Effects on the Infant Microbiome

Although most studies report no differences in the microbiome of VD and CSD neonates in the first days of life, evidence is compelling that differences begin to develop shortly thereafter and persist for weeks or months (Table 1).

Bäckhed and colleagues sampled the gut microbiota of 98 infants, 15 of which were delivered by CS, at 4 days, 4 months, and 1-year post-delivery (51). Although a high rate of mother-to-infant transmission of bacteria was observed, regardless of mode of delivery, this transmission was partially compromised in infants born by CS. Infants born vaginally were enriched for *Bacteroides* spp. across all time points and *Parabacteroides* spp. at 4 days and 4 months. CSD infants harbored higher relative levels of *Clostridium* spp. at 4 months and 1 year. Apart from these trends, the infant gut appeared to be highly dynamic throughout the first year of life.

Similar findings were reported by Penders et al. in their broad study of over 1,000 infants at 1 month of age (52). The authors observed significantly lower levels of *Bacteroides* spp. in CSD infants, and also found that CSD infants were more often colonized by *Clostridium difficile*. The biological significance of this observation is currently unclear, although it could be interpreted to be precursor of dysbiosis. Azad et al. further corroborated these finding, reporting significantly lower levels of *Bacteroides* spp. in CSD infants at 4 months of age (50). This study additionally identified a decrease in *Escherichia*–*Shigella* spp. in CSD infants.

Sakwinska et al. investigated the nasal and stool microbiomes of infants born vaginally (*n* = 34) and by CS (*n* = 8) at 3 days and 3 weeks of life, and compared their composition to maternal vaginal, skin, and rectal samples (49). They reported that the stool of CSD infants at week 3 was more similar to the maternal skin than that of VD infants. In their study, only VD, exclusively breastfed infants had gut microbiota dominated by *Bifidobacteria* spp. (which is found abundantly in breast milk and the infant gut and is believed to confer health benefits to the host).

There has been one recent study that challenged our current understanding of the CS delivery microbiome. Chu and colleagues prospectively recruited 81 mother-infant pairs (37). They reported that, after adjusting for confounders, mode of delivery had no impact on microbial community structure or function over multiple body sites (skin, oral, nares, stool) at 6 weeks of age.

In general, initial differences between VD and CSD infants’ microbiomes appear to resolve after solid foods are introduced. In Wampach et al.’s study, which expanded our current understanding of the infant microbiome into the kingdoms of archaebacteria and fungi, differences between CSD and VD infants became less pronounced after day 150 (31). The authors’ suggested that this change was driven by the introduction of solid foods. This observation is supported by several other studies which have found that introduction of solid food sparks a rapid change in the infant gut microbiome toward a more mature gut microbiome, minimizing any initial differences associated with mode of delivery (53, 54). A systematic review of seven studies concluded that there was a significant difference in the microbiota of CSD infants up until 3 months of age, but that this difference disappears after 6 months (55). Reduced levels of *Bacteroides* spp. in CSD infants may persist up to 4 weeks post-weaning (53).

Despite this post-weaning convergence, a transient difference in the infant gut microbiome early in life may nevertheless be biologically significant. The early-life gut microbiome plays a critical role in the development of the immune system, which impacts upon neurodevelopment (56, 57) and risk of later-life metabolic and immune-mediated diseases (15, 58, 59). Thus, it is vital to clarify the true impact of mode of delivery on the early-life microbiome. In particular, when considering potential treatment and management strategies, there is a need to elucidate whether differences seen in CSD infants are due to lack of passage through the birth canal or due to other factors associated with CS delivery.

## Confounding Factors

There are numerous maternal and medical factors that confound the interpretation of studies of the microbiome and health outcomes of CSD infants. Here, we will describe the major confounding factors in such studies.

### Antibiotics

Antibiotics are powerful disrupters of the developing gut microbiome. Depending on the type, dose, duration, and timing of administration (60), antibiotics can have profound effects on the development of the neonatal and/or infant microbiome. All mothers delivering by CS are administered intrapartum antibiotic prophylaxis (IAP), as is routine for any type of surgery. In some countries, IAP is administered after the cord is clamped, minimizing direct antibiotic exposure of the neonate. In others, antibiotics are given prior to commencement of surgery. For those antibiotics which cross the placenta, IAP will likely have a devastating effect on the neonate’s microbiota, although it should be born in mind that the microbial DNA will remain unaltered even if the bacteria are dead. Hence, microbiome studies may fail to appropriately recognize the effects of antibiotic exposure within the first day or so of life. Mothers delivering vaginally are not routinely administered antibiotics, with the notable exception of those who are vaginally colonized with Group B *Streptococcus* (GBS). Overall, rates of intrapartum antibiotic use are low in vaginally delivering mothers (61).

The effects of IAP on the neonatal gut microbiome are well-described (62–67). In particular, maternal IAP has been associated with decreased bacterial diversity in the neonatal gut, as well as a decrease in the relative abundance of Actinobacteria, Bacteroidetes, *Bifidobacteria* spp., *Bacteroides* spp., *Parabacteroides* spp., *Atopobium* spp., and *Lactobacillus* spp., and an increase in the relative abundance of Proteobacteria, Firmicutes, Enterobacteriaceae, *Enterococcus* spp., and *Clostridium* spp. These perturbations to the neonatal gut microbiome directly overlap with those observed in CSD infants (Table 2). Importantly, these perturbations are accompanied by a delay in the production of immunomodulatory short chain fatty acids (62).

**Table 2:** Genera of bacteria reported to be differentially abundant in [Bäckhed et al. (51), Sakwinska et al. (49), Penders et al. (52), Tun et al. (68), and Azad et al. (50)] cesarean section delivered (CSD) infants compared to vaginally delivered (VD) infants across five studies.

Genus	Intrapartum antibiotic prophylaxis (IAP)	Cesarean section delivery						
		4 days	3 weeks	1 month	4 months			1 year
		
		Bäckhed et al.	Sakwinska et al.	Penders et al.	Tun et al.	Bäckhed et al.	Azad et al.	Bäckhed et al.
*Actinomyces*		↓						
*Aggregatibacter*		↑						
*Anaerococcus*								↑
*Anaerostipes*						↑		
*Bacillus*					↑			
*Bacteroides*	↓(63)^a^, (64)^b^, (66)^d^	↓	↓	↓		↓	↓	↓
*Bifidobacterium*	↓(64)^b^, (65)^b^, (67)^c^		↓	↓				
*Bilophila*						↓		
*Blautia*								↑
*Brevundimonas*						↑		
*Butyrivibrio*								↑
*Capnocytophaga*								↑
*Citrobacter*								↑
*Clostridium*	↑(63)^a^ (67)^c^					↑		↑
*Collinsella*	↓(63)^a^	↓						
*Comamonas*					↓			
*Cronobacter*								↑
*Deinococcus*					↑			
*Delftia*						↑		
*Enterobacter*						↑		
*Enterococcus*	↑(63)^a^					↑		
*Escherichia*–*Shigella*	↑(64)^b^	↓					↓	
Eubacterium					↑			
*Faecalibacterium*	↑(63)^a^				↑			
*Granulicatella*						↑		
*Haemophilus*		↑						
*Klebsiella*			↑					
*Lactobacillus*	↓(67)^c^							
*Macrococcus*						↑		
*Parabacteroides*	↓(63)^a^	↓				↓		
*Paraprevotella*								↑
*Propionibacterium*					↓			
*Proteus*						↑		
*Providencia*						↑		
*Pseudomonas*					↓			
*Rothia*	↑(63)^a^	↑						
*Staphylococcus*		↑				↓		
*Stenotrophomonas*					↑			
*Streptococcus*	↑(63)^a^ (64)^b^	↑						
*Veillonella*	↑(63)^a^ ↓(64)^b^	↑						

*^a^Antibiotic not specified*.

*^b^Ampicillin*.

*^c^Ampicillin and gentamycin*.

*^d^Various antibiotics*.

*↑ indicates a significant increase in relative abundance in CSD infants compared to VD infants. ↓ indicates a significant decrease in relative abundance in CSD infants compared to VD infants. These disturbances are compared with disturbances associated with IAP as reported in six studies (62–67)*.

Perinatal antibiotic use has also been associated with increased risk of developing numerous morbidities (69), including asthma (70–73), allergies (74, 75), and obesity (76). It is vital to consider antibiotic use, in particular the specific types of antibiotics used and the timing of administration, when comparing the microbiome composition and health outcomes of CSD and VD infants, as differences in these infants may be directly attributable to differences in antibiotic exposure (Table 2).

### Labor

The absence of labor in mothers delivering by elective CS creates a completely different biochemical landscape in the maternal body. Specifically, labor causes changes in levels of endocrine, inflammatory, and contractile factors. These changes might influence the maternal microbiome or the establishment of the neonatal microbiome. Additionally, labor is often accompanied by rupture of the fetal membranes, exposing the fetus to maternal vaginal bacteria. Accordingly, previous studies have demonstrated a difference in the neonatal microbiome of women delivering by elective CS (no labor) compared to those delivering by emergency CS (labor) (50).

Both maternal and fetal cytokines influence the developing fetal immune system. Malamitsi-Puchner et al. investigated cytokine concentrations in maternal, fetal, and neonatal blood after vaginal delivery (labor) and elective Cesarean delivery (no labor) (77). They found that vaginal delivery was associated with an increase in circulating cytokines up to 4 days post-partum, probably due to the consequences of labor, which is known to be pro-inflammatory and did not occur in the elective CS group.

Differences in levels of inflammatory and immune-mediating cytokines in CSD infants (due to absence of labor) may account for some of the observed differences in neonatal microbiome colonization and health outcomes. This possibility is supported by a number of studies showing that elective Cesarean delivery, but not emergency Cesarean delivery (where often the labor process has commenced), is associated with an increased risk of asthma (78), celiac disease (79), and psychosis (80). Interestingly, while fecal bacterial richness and diversity are lower among infants born by elective Cesarean delivery compared to those born vaginally, they are also higher in infants born by emergency Cesarean delivery compared to those born vaginally (50). This phenomenon remains to be explained.

### Breastfeeding

Breast milk contains a number of important nutritional components, as well as bacteria, which have repeatedly been shown to markedly influence the infant gut microbiome (52, 63, 81–86); this, in turn, modifies host gene expression and immune development *via* the production of bacterial metabolites and systemic exposure to bacterial products (84–86). Source tracking studies have shown that 27% of an infant’s gut microbiota is vertically derived from its mother’s breast milk, while an additional 10% is sourced from the skin around the areola (87). Additionally, breast milk is rich in prebiotics, such as human milk oligosaccharides, which are metabolized by certain members of the gut microbiota and modify the composition of the infant gut microbiome and metabolome (88).

Cesarean section deliveries are associated with increased risk of a range of sub-optimal breast feeding parameters. These include a delay in breast feeding (89), a shortened duration of breastfeeding (90), sub-optimal breastfeeding behavior (89), and a reduced volume of breast milk consumption in the first 5 days of life (91). Given the microbial and immune modulatory properties of human breast milk, it is highly likely that differences in feeding practices between CSD and VD infants might account for some of the microbial and epidemiological differences observed in these groups. This phenomenon would also explain, in part, why differences in infant gut microbiome diminish with weaning.

### Maternal Obesity

Maternal obesity is a known risk factor for Cesarean delivery in both developed and developing countries; the OR for obese women having a CS delivery compared with women of normal weight is 2.01–2.36 (68, 92–97). High maternal BMI is associated with an increased risk for failed labor, which in turn increases labor duration and ultimately leads to CS delivery (68, 92, 98).

Obesity and high-fat diets have repeatedly been correlated with aberrations to the gut microbiome in humans (99–102). Maternal obesity alters the maternal gut microbiome during pregnancy (103, 104), and the milk microbiome during lactation (105, 106), both of which are likely to influence both pre- and post-natal microbiome colonization patterns and immune development in the offspring. Importantly, Chu et al. demonstrated an association between maternal high-fat diet and distinct changes in the neonatal gut microbiome at birth, which persisted through 4–6 weeks of age (107).

Mother-to-child transmission of obesogenic microbes continues to disrupt microbiome patterns into early childhood. Galley et al. found that the gut microbiomes of toddlers born to obese mothers of high socioeconomic status (SES) clustered away from those of toddlers born from lean high SES mothers (108). In particular, children born to obese mothers had differences in abundances of *Faecalibacterium* spp., *Eubacterium* spp., *Oscillibacter* spp., and *Blautia* spp., all of which have been correlated to diet and body weight in previous studies.

Interestingly, a recent study published by Mueller et al. reported that maternal obesity-associated differences in the neonatal microbiome are dependent on delivery mode (109). They found that the fecal microbiota of VD infants (*n* = 18) was disturbed by maternal obesity (*n* = 5) on day 2 of life. This association was not evident in CSD neonates (*n* = 56, 26 of which were obese or overweight). While the authors suggest that their results indicate that an altered microbiome is transferred to offspring of obese mothers during vaginal delivery, it is also possible that their results reflect differences in IAP between groups. Importantly, the meconium microbiota at day 2 of life (often the time of the first bowel movement) is likely to reflect the fetal gut contents *in utero*, which is likely to be influenced by maternal diet and health status. Thus, we would expect to see differences based on maternal pre-pregnancy BMI—regardless of delivery mode. It is possible that antibiotic administration during pregnancy (*n* = 11 in the CSD group, none reported in the VD group) and delivery (no antibiotic use during delivery reported in the VD group) could mask these differences.

Similarly, Tun et al. found that maternal obesity perturbs the infant microbiome in a delivery mode-dependant manner (68). They reported that VD infants (*n* = 708) born to overweight or obese mothers had a statistically higher abundance of several genera (e.g., *Bacteroides* spp., *Megasphaera* spp., *Blautia* spp., and *Oscillospira* spp.) and reduced abundance of others (e.g., *Haemophilus* and *Veillonella*), compared to those born from normal weight mothers. Among infants born after emergency CS delivery, *Coprococcus* spp. and *Ruminococcus* spp. were more abundant in infants born to overweight or obese mothers than normal weight mothers. Importantly, CS delivery was found to be associated with a twofold increase in risk of obesity in the infant, but this association disappeared after adjustment for co-variables, such as maternal obesity and IAP.

Transmission of aberrant or obesogenic microbiota from obese mothers could potentially explain some of the microbial and metabolic differences reported in infants born by CS vs. those born vaginally. However, this potential mechanism remains unproven, as the ability to induce an obese phenotype *via* transfer of obesogenic microbiota has only been demonstrated in animal models thus far (102).

### Gestational Age at Delivery and Neonatal Intensive Care Unit (NICU) Exposure

Rates of CS delivery increase with decreasing gestational age at delivery (110). Preterm infants differ from their full-term counterparts in terms of their gut microbiota (38, 111–113), immune development (114, 115), and health outcomes (116–118). Arboleya et al. reported that aberrations to the gut microbiota of preterm infants in the first 3 months of life are coupled with a difference in metabolically active short chain fatty acid levels in stool. Although not tested in this particular study, it would be expected that such differences would result in altered immune development among preterm infants.

Preterm infants also experience different nutritional and environmental exposures after birth. The NICU environment is likely to influence the microbiome, so duration of residence and the environmental microbiome of the unit are likely to have a significant impact (119). Infants residing in the NICU also experience differences in nutrition (formula and donor breast milk are common) and antibiotic administration (120). Thus, gestational age and the influence of exposure to the NICU must be accounted for in studies comparing CSD and VD infants.

### Inter-Individual Variation

The human microbiome is tremendously variable, both in terms of inter-individual and intra-individual variation (121–126). Thus, any study wishing to define microbial differences in two populations must take into account baseline variability in individual microbiomes, as well as temporal changes within an individual. Studies that compare the microbiomes of infants born by CS or vaginal delivery must have sufficient power to account for variation in the maternal microbiome, as this is likely to exert a large influence on an infant’s microbiome through breastfeeding and physical contact. Large cohorts are thus required with the ability to control variables, such as home environment, presence of pets, and exposures to other microbiome-altering factors including hygiene and maternal/infant diet.

## Postnatal Vaginal Seeding

As discussed above, although the data are supportive of a difference in the early-life microbiome of infants born *via* CS vs. vaginal delivery, the evidence that this is due to the mode of delivery (i.e., differences in exposure to vaginal microbiota during birth) is unconvincing and lacking in critical data. Nevertheless, the perception among the public and medical health professionals alike is that CS delivery deprives the infant of exposure to vaginal microbiota and this leads to neonatal dysbiosis and increased risk of poorer health outcomes. As a consequence, attempts have been made to correct the problem—even though the “problem” may not exist and the benefits of exposure to any individual bacterial species have not been determined.

The widely cited and influential study of Dominguez-Bello and colleagues sought to address the lack of vaginal bacterial exposure in CSD neonates using a practice that they termed “vaginal seeding” (127). Vaginal seeding involves the use of a gauze swab to transfer maternal vaginal fluid, and the microbes contained therein, to a neonate immediately following CS delivery. To test the effectiveness of this procedure, the authors recruited 18 mothers delivering either vaginally (*n* = 7) or by CS (*n* = 11). Four of the CSD neonates were exposed to their mothers’ vaginal fluid following birth. The authors reported a partial restoration of the neonatal microbiome (mainly the skin and oral microbiome, less so the gut microbiome) in these four infants after vaginal seeding, with exposed neonates exhibiting microbiomes similar to those of their VD counterparts.

This study had several major weaknesses. Importantly, all mothers delivering by CS received IAP, while only one mother who delivered vaginally was exposed to antibiotics. Additionally, all CS deliveries were elective (no labor), a limitation which the authors themselves acknowledged. It is also important to note that differences in maternal pre-pregnancy BMI and gestational weight gain were not accounted for in this study. The limitation of the small sample size in this study was exacerbated by the fact that there were several missing samples at each time point. Given the high level of baseline inter-individual variation that was evident, the perceived differences in the vaginal fluid exposed group (*n* = 4) may be purely coincidental and the data are very unconvincing. In fact, swabs of the neonate’s hands immediately following birth revealed that only 3 of the 7 VD neonates had skin microbiomes dominated by *Lactobacillus*, while 1 of the 2 CSD neonates sampled at birth also had a *Lactobacillus*-dominated skin microbiome, as did all three of the swabbed neonates sampled at birth. This is in line with previous reports from Martin et al., showing that passage through the vagina rarely transfers *Lactobacillus* to the infant (47). Remarkably, the primers used in this study were not reported, making it impossible to judge the bacterial coverage of the study.

The premise of this study was based on the assumption that the health consequences and microbial disruptions of CS delivery are due to a lack of exposure to vaginal microbes during delivery. As described in this review, there are a multitude of factors that might contribute to the Cesarean delivery phenotype, and very little evidence to support the theory that exposure to maternal vaginal microbes during delivery is important. Despite the absence of strong supporting evidence for this concept, the notion that replicating the vaginal seeding process will help to mitigate the perceived risks of CS delivery by restoring the natural microbial homeostasis of the infant has gained considerable traction. Alarmingly, the practice of vaginal seeding has become main-stream in some areas, and is often performed without the knowledge or guidance of health care professionals. This practice carries a serious risk of transferring opportunistic pathogens (including viruses and fungi) to newborns which might be asymptomatic in the mother (most notably, GBS) (128). We agree with and support the recent guidelines from the American College of Obstetricians and Gynecologists and the Danish Society of Obstetrics and Gynecology with respect to vaginal seeding in the management of CS delivery (129, 130).

If evidence is eventually provided that vaginal seeding does play a role in the formation of the early-life microbiome, it may be more beneficial to employ the more controllable and pharmacologically safer method of probiotic administration to help promote normal neonatal microbiota. Probiotics are already being trialed to correct dysbiosis preceding NEC in preterm neonates with some success (131, 132), and probiotic formulations of vaginal bacteria are already commercially available. If such an approach is shown to be warranted and potentially beneficial, it could have significant safety and therapeutic advantages over the maternal vaginal seeding procedure trialed by Dominguez-Bello and colleagues.

## Conclusion

There is certainly a transient difference in the gut microbiota of infants born by Cesarean delivery compared to their VD counterparts. While this difference appears to be corrected after weaning, it may have lifelong impacts on the development of the immune system. This might underpin the increased incidence of asthma, allergies, and autoimmune diseases seen in CSD infants later in life. However, given the numerous and significant confounding factors present in studies comparing the microbiota after CS and vaginal delivery, it is impossible to say with any certainty that it is the act of delivering vaginally itself which confers this optimal microbiota, or what species/genera of bacteria might be responsible. Differences in antibiotic administration, labor onset, maternal body weight and diet, gestational age, and breastfeeding frequency and duration undoubtedly contribute to differences observed between CSD and VD infants. Further, it is likely that differences between CSD and VD infants do not develop until several days after birth. Given recent evidence that infant microbiome colonization begins *in utero*, it may be that the importance of “bacterial baptism” of vaginal birth has been significantly over-estimated.

There is a high level of conflicting evidence surrounding this topic. To truly assess the impact of delivery mode on the neonatal microbiome, large scale studies are needed in which mothers delivering by CS are paired with BMI-matched vaginally delivering controls who also receive the same intrapartum antibiotics and who follow similar breastfeeding patterns, combined with strain-specific high-resolution microbiome studies to confirm microbial sources of origin. Although numerous studies have demonstrated an association between CS delivery and altered microbiome establishment, no studies have confirmed causality; the substantial differences in the methodology that exists between studies, including lack of appropriate controls and species-level resolution, further confounds our ability to define this apparent association. Health practitioners should not bow to popular pressure to perform vaginal seeding in the absence of data on need, effectiveness, and appropriate protocols for ensuring safety.

## Author Contributions

LS researched and wrote the manuscript. MP and JK contributed to discussion and idea development and critically edited the manuscript.

## Conflict of Interest Statement

The authors declare that the research was conducted in the absence of any commercial or financial relationships that could be construed as a potential conflict of interest.

## Abbreviations

CSD, cesarean section delivered; DNA, deoxyribonucleic acid; GBS, group B *Streptococcus*; IAP, intrapartum antibiotic prophylaxis; MRSA, methicillin-resistant *Staphylococcus aureus*; NEC, necrotizing enterocolitis; NICU, neonatal intensive care unit; OECD, organization for economic co-operation and development; PCR, polymerase chain reaction; qPCR, qualitative polymerase chain reaction; RNA, ribonucleic acid; rRNA, ribosomal ribonucleic acid; RT-qPCR, real-time qualitative polymerase chain reaction; SES, socioeconomic status; VD, vaginally delivered.
